# Cavitary Pulmonary Infarction Mimicking Malignancy: A Diagnostic Challenge Resolved by Conservative Management

**DOI:** 10.7759/cureus.96096

**Published:** 2025-11-04

**Authors:** Ahmed Elgohary, Miles Chapman, Aleksandra Plichta, Dionyssios Malamis

**Affiliations:** 1 Respiratory, East Kent Hospitals University NHS Foundation Trust, Margate, GBR; 2 Cardiology, East Kent Hospitals University NHS Foundation Trust, Margate, GBR; 3 Radiology, East Kent Hospitals University NHS Foundation Trust, Margate, GBR

**Keywords:** cavitary lung lesion, conservative medical management, ct pulmonary angiogram (ctpa), pulmonary cavitation, pulmonary embolism, pulmonary infarction, therapeutic anticoagulation

## Abstract

Cavitating pulmonary infarction is an uncommon complication of pulmonary embolism (PE) that can closely mimic lung malignancy or necrotising infection on imaging. We report the case of a 72-year-old man with a history of heavy smoking who presented with subacute cough, dyspnoea, anorexia, and weight loss. Imaging revealed bilateral PE with right heart strain and a large, thick-walled cavitary lesion in the right lower lobe. Extensive microbiological, cytological, and autoimmune testing did not identify infection, vasculitis, or malignancy. Following multidisciplinary team discussion, the patient was managed conservatively with anticoagulation and a short empirical antibiotic course. Serial imaging demonstrated complete resolution of the cavity and emboli over eight months, with full clinical recovery. This case underscores the importance of correlating imaging findings with vascular anatomy, maintaining a high index of suspicion for pulmonary infarction in patients with coexisting PE, and using a structured, multidisciplinary approach to avoid unnecessary biopsy or surgery when conservative management is likely to be curative.

## Introduction

Cavitary pulmonary infarction is a rare but clinically significant complication of pulmonary embolism (PE), occurring in fewer than 5% of cases [1,2]. Although PE is a common and potentially life-threatening condition, cavitation of the infarcted lung tissue remains an uncommon complication, reflecting the limited bronchial collateral circulation in affected regions [2]. The formation of a thick-walled pulmonary cavity can closely mimic other conditions such as squamous cell carcinoma, necrotising pneumonia, mycobacterial or fungal infections, and granulomatous vasculitis, often prompting extensive and invasive diagnostic evaluations [3-6].

Recognising cavitation as a consequence of infarction is therefore crucial to avoid unnecessary biopsy, surgical resection, or prolonged antimicrobial therapy. Most pulmonary infarctions are small and resolve spontaneously; however, large cavitary infarctions are rare and pose significant diagnostic and management challenges [7,8].

This case is distinctive due to the unusually large cavitary lesion that developed following acute PE and its complete resolution with conservative, non-invasive management. It underscores the importance of careful clinico-radiologic correlation, systematic exclusion of alternative causes, and multidisciplinary discussion to ensure accurate diagnosis and optimal patient outcomes.

## Case presentation

A 72-year-old man presented with a one-month history of non-productive cough, exertional dyspnoea, anorexia, and unintentional weight loss of 6.4 kilograms (kg) (Table 1). His past medical history included rheumatoid arthritis treated with methotrexate, hypothyroidism, and stage three chronic kidney disease (CKD). He had a 60 pack-year smoking history but was functionally independent.

**Table 1:** Case timeline CTPA: CT pulmonary angiogram; BAL: bronchoalveolar lavage

Timepoint	Clinical event
~1 month prior	Onset of cough, dyspnoea, anorexia, and 6.4 kg weight loss
Day 0	Hospital presentation; CTPA shows bilateral pulmonary emboli and lung cavity
Day 1–7	Intravenous antibiotics and oral anticoagulation initiated
Day 7	Auto-immune screen negative
Day 10	Bronchoscopy performed; BAL cultures and cytology negative
Day 14	Patient discharged on conservative oral anticoagulation
Month 1	Multidisciplinary team (MDT) review supports conservative management
Month 8	Repeat CTPA confirms full resolution of cavity and emboli

On admission, he was tachypnoeic at 29 breaths per minute, tachycardic at 118 beats per minute, normotensive, afebrile, and had oxygen saturation of 96% on room air. Examination was otherwise unremarkable apart from mild abdominal tenderness and bilateral pitting oedema.

Initial laboratory results showed WBC 15.6 × 10⁹/L, CRP 306 mg/L, and D-dimer 6.70 µg/mL (Table 2).

**Table 2:** Laboratory investigations ↓: below reference range; ↑: above reference range CRP: C-reactive protein; eGFR: estimated glomerular filtration rate; FEU: fibrinogen-equivalent units; AAFB: acid-alcohol fast bacilli; ANCA: antineutrophil cytoplasmic antibody; ANA: antinuclear antibody

Investigation/test	Result	Reference range	Units
Haemoglobin	124 ↓	130–180	g/L
White blood cells	15.6 ↑	4.0–11.0	×10⁹/L
Neutrophils	13.3 ↑	2.0–7.5	×10⁹/L
C-reactive protein (CRP)	306 ↑	<5	mg/L
Creatinine	167 ↑	60–110	µmol/L
eGFR	35 ↓	>60	mL/min/1.73 m²
D-dimer	6.70 ↑	<0.5	mg/L FEU
Respiratory sputum	Purulent; normal flora only	—	—
AAFB/Mycobacteria culture (6 weeks)	Negative	Negative	—
Mycoplasma pneumoniae IgM	Negative	Negative	—
Legionella and Streptococcus pneumoniae urinary antigen	Negative	Negative	—
Bronchial washings	Normal flora only; galactomannan negative	—	—
ANCA/rheumatoid factor/ANA	Negative	Negative	—
Histology (bronchial washing and brushing)	Reactive bronchial epithelial cells, macrophages, neutrophils; no malignant cells	—	—

**Figure 1:**
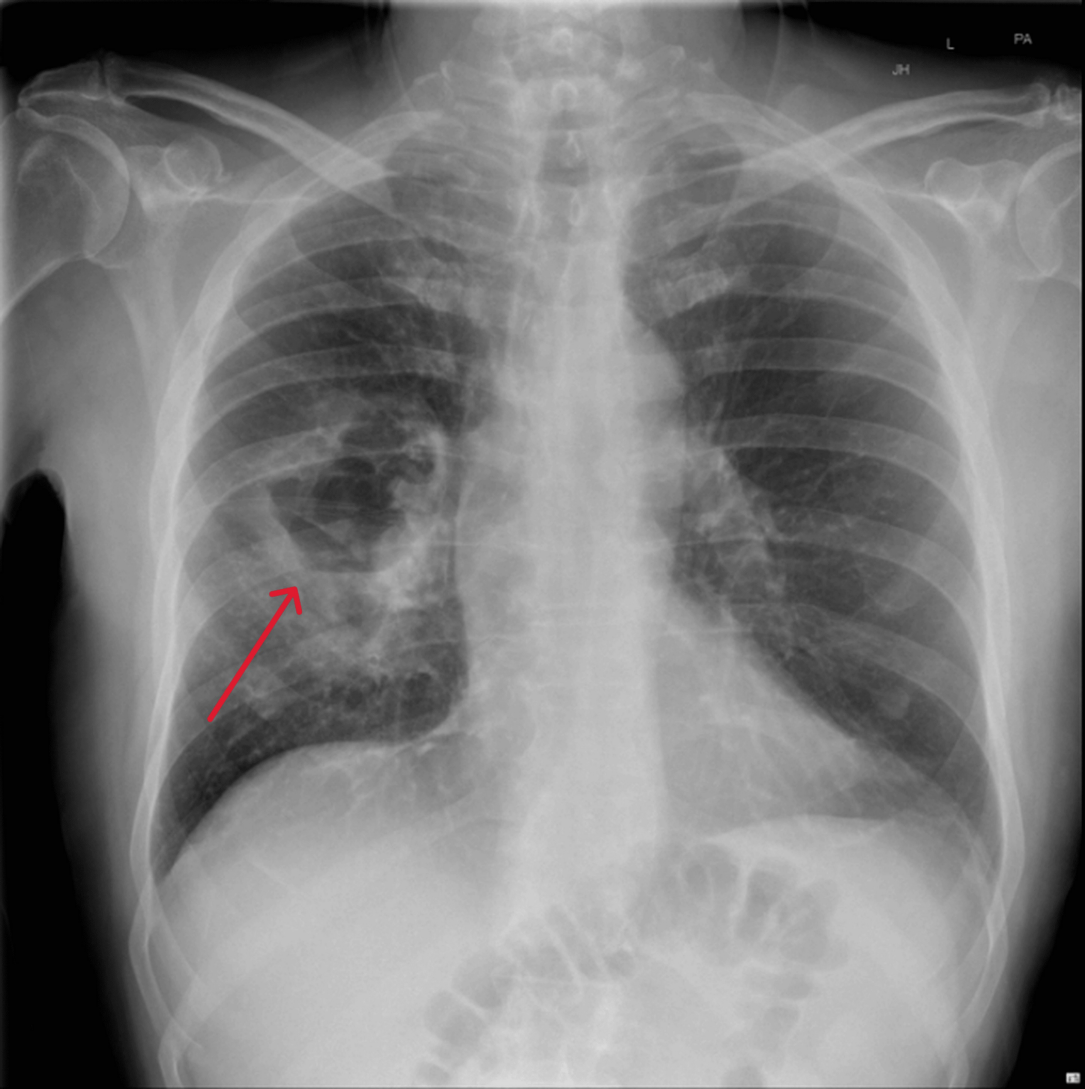
Chest x-ray (CXR) on admission The red arrow points toward the cavitating lesion seen in the right mid-zone.

**Figure 2:**
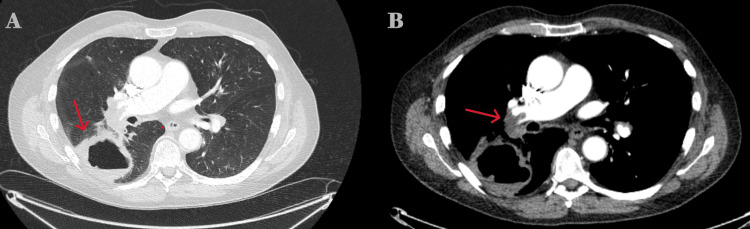
CT pulmonary angiogram (CTPA) images on admission The red arrow in A shows the cavitating lesion, while the red arrow in B shows the pulmonary embolus in direct correlation with the lesion.

Chest radiography (CXR) revealed a right mid-zone cavity (Figure 1). CT pulmonary angiography (CTPA) confirmed bilateral PE with right heart strain and identified a 7.5 cm thick-walled cavitary lesion in the posterior basal segment of the right lower lobe, abutting the pleura (Figure 2).

He received empirical intravenous co-amoxiclav for seven days, followed by 14 days of oral therapy, and anticoagulation with apixaban 10 mg twice a day for one week, followed by 5 mg twice daily thereafter for six months.

**Figure 3:**
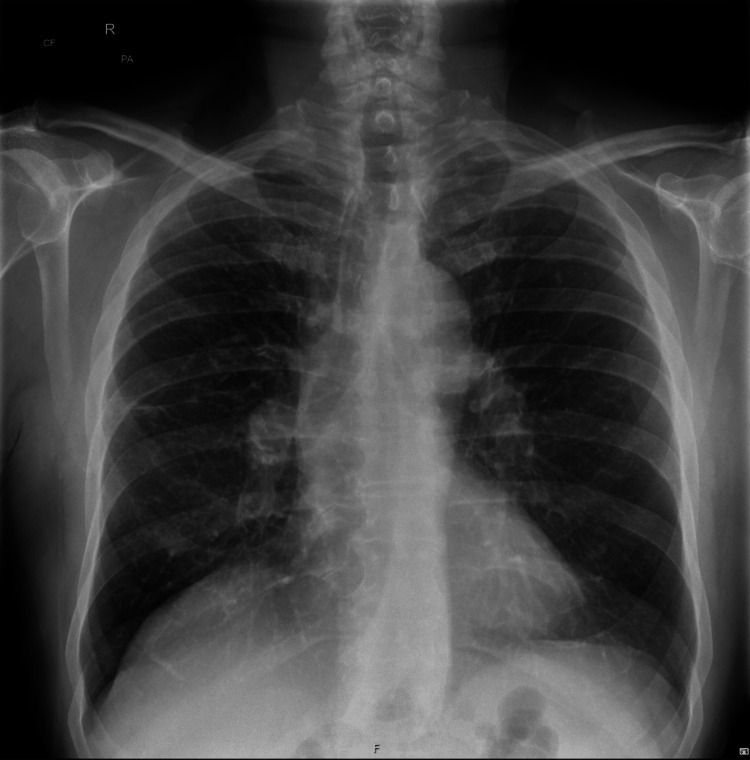
Chest x-ray (CXR) eight months later showing complete resolution of the cavitating lesion

**Figure 4:**
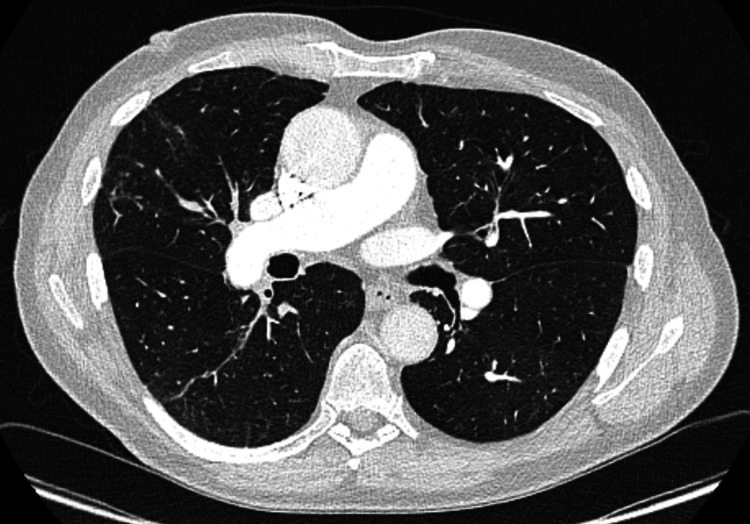
CT pulmonary angiogram (CTPA) eight months later showing complete resolution of the cavitating lesion

Further investigations were then performed to exclude alternative diagnoses. Bronchoscopy showed no endobronchial lesion, and bronchoalveolar lavage was negative for bacteria, mycobacteria, and fungi, whereas his cytology demonstrated only reactive epithelial changes (Table 2). Autoimmune screening, including ANCA and ANA, was negative (Table 2). In view of the absence of infection, malignancy, or vasculitis, and following multidisciplinary team review, a decision was made to manage the patient conservatively with continued anticoagulation and interval imaging. At eight-month follow-up, repeat CXR (Figure 3) and CTPA (Figure 4) showed complete resolution of the cavitary lesion and pulmonary emboli. Inflammatory markers had normalised, and the patient reported full recovery.

## Discussion

Cavitating pulmonary lesions in older patients with constitutional symptoms and a heavy smoking history commonly raise concern for lung malignancy or necrotising infection [3-5]. In this case, the differential diagnosis included squamous cell carcinoma, bacterial or fungal abscess, tuberculosis, and granulomatous vasculitis. The patient’s anorexia, weight loss, and elevated inflammatory markers initially supported these considerations. However, the acute presentation, absence of fever, and negative bronchoscopy, cytology, and microbiological cultures argued against infection or malignancy. Autoimmune testing (ANCA, ANA, rheumatoid factor) was negative, excluding vasculitic and rheumatoid causes [6].

CT pulmonary angiography (CTPA) demonstrated a large, peripheral, pleural-based cavitary lesion precisely corresponding to a segmental pulmonary artery occlusion, establishing the diagnosis of cavitary pulmonary infarction. The anatomic correlation and absence of a discrete soft-tissue mass were decisive in differentiating it from neoplastic or infective causes [3-6].

Cavitary pulmonary infarction occurs in fewer than 5% of pulmonary embolism (PE) cases and is typically associated with large infarcts, advanced age, and right heart strain [1,2,7]. CT findings classically demonstrate wedge-shaped peripheral consolidations that may cavitate as necrosis develops [2,8]. The early development of a large cavity in this patient likely reflected acute necrosis of a substantial infarct rather than delayed cavitation, an infrequently reported phenomenon [2].

Management was guided by literature supporting conservative treatment with guideline-directed anticoagulation once infection and malignancy are excluded [2,8]. The patient received apixaban 10 mg twice daily for seven days, followed by 5 mg twice daily, consistent with British Thoracic Society PE guidelines [8]. A short empirical course of co-amoxiclav was administered pending culture results. Progressive clinical improvement and complete radiologic resolution over eight months confirmed the effectiveness of this conservative, non-invasive strategy.

This case highlights the importance of integrating vascular anatomy, imaging, and targeted laboratory evaluation in assessing cavitary lung lesions. Recognising pulmonary infarction as a potential cause can prevent unnecessary invasive procedures and allow safe, evidence-based management.

## Conclusions

Cavitary pulmonary infarction should be considered in patients presenting with a solitary cavitary lung lesion and concurrent pulmonary embolism, especially when microbiological, cytological, and autoimmune investigations are negative. Correlation of imaging findings with vascular anatomy is critical for diagnosis. Conservative management with anticoagulation and careful follow-up can result in complete resolution and prevent unnecessary invasive interventions.
